# Supplementary material for: Vitrification Using Soy Lecithin and Sucrose: A New Way to Store the Sperm for the Preservation of Canine Reproductive Function
Source: Animals (Basel). 2020 Apr 9;10(4):653. doi: 10.3390/ani10040653 (PMC7222707; doi:10.3390/ani10040653)
Supplement: Supplementary file 1 [file animals-10-00653-s001.pdf]

Comparison of different techniques for assessing semen concentration

Supplementary table 1: Mean values ± standard deviation and coefficient of variation (CV) of semen concentration (× 10^6/mL) obtained with different counting techniques—Neubauer chamber, spectrophotometer (SpermaCue) and CASA system.

Different conc. measurements × 10^6/ml	Ejaculates				
	1	2	3	4	CV (%)
Neubauer chamber	295 ± 22.9	413 ± 18.9	241 ± 13.2	250 ± 17.3	6.16 ± 1.41
SpermaCue	278 ± 9.3	413 ± 12.4	238 ± 11.2	274 ± 11.7	3.83 ± 0.79
CASA	336 ± 13.2	457 ± 15.3	287 ± 14.8	314 ± 14.2	4.24 ± 0.78

Within the same columns, different superscripts show significant difference at $p < 0.05$.

Optimization of the best soy lecithin concentration added to the basic extender

Supplementary table 2: Basic semen parameters (mean ± SD) were evaluated after devitrification and compared to those in the basic extender alone.

Semen parameters (%)	Basic extender	Soy lecithin			
		1 %	2 %	3 %	4 %
Motility	3.5 ± 1.1[a]	47.6 ± 7.5[b]	37.2 ± 6.7[c]	32.4 ± 8.9[c]	20.9 ± 6.1[d]
Progressive motility	1.9 ± 1.0[a]	39.6 ± 2.5[b]	22.7 ± 3.7[c]	16.7 ± 2.8[c]	6.6 ± 0.28[a]
Morphologically normal	59.4 ± 8.5[a]	71.4 ± 7.2[b]	66.8 ± 10.5[b]	63.4 ± 10.3[ab]	52.5 ± 9.5[a]
Viability	35.9 ± 1.9[a]	59.2 ± 2.8[b]	54.5 ± 2.2[b]	39.7 ± 2.1[a]	31.6 ± 1.1[a]

Within the same row, different superscripts show significant difference at $p < 0.05$. Basic extender in the preliminary study was extender C (control extender that contained 0.25 M sucrose).
